# Diagnosis and treatment of multisystem amyloidosis associated with *SGPL1* mutation: A case report and review of the literature

**DOI:** 10.1097/MD.0000000000046653

**Published:** 2026-01-02

**Authors:** Yunfen Chen, Yue Liao, Mingxia Ding, Yinghua Chen, Ya Chen, Bangneng Yu, Xiuying Fan, Xuedong Yi, Yihuai He, Yawen Luo

**Affiliations:** a Department of Infectious Diseases, Affiliated Hospital of Zunyi Medical University, `Zunyi, Guizhou Province, China; b Department of Nephrology, Bozhou District People’s Hospital, Zunyi, Guizhou Province, China; c Guizhou Provincial Key Laboratory of Cell Engineering, Zunyi, Guizhou Province, China; d Pharmacy Department, Affiliated Hospital of Zunyi Medical University, Zunyi, Guizhou Province, China.

**Keywords:** amyloidosis, monoclonal IGA-κ protein, nephrotic syndrome, *SGPL1* gene mutation, sphingosine-1-phosphate

## Abstract

**Rationale:**

Amyloidosis is a rare, clinically heterogeneous disease, which makes its diagnosis difficult. The relationship between amyloidosis and gene mutations is insufficiently understood. We report a case of sphingosine-1-phosphate lyase 1 (*SGPL1*) mutation-related amyloidosis, and review the related literature.

**Patient concerns:**

A 53-year-old man was admitted to our hospital with a 5-month history of renal dysfunction and abdominal distension, aggravated since 2 days. Five months ago, the patient was diagnosed with nephrotic syndrome, with liver dysfunction and abdominal distension. His symptoms improved after treatment with methylprednisolone sodium succinate, dipyridamole, hydroxychloroquine sulfate, and ursodeoxycholic acid. Two days ago, his abdominal distension worsened, and was not associated with eating, acid reflux, heartburn, fatigue, or poor appetite. A physical examination showed periumbilical ecchymosis and hepatic enlargement. Blood biochemistry showed kidney dysfunction, dyslipidemia, increased alkaline phosphatase and glutamyltransferase, and decreased albumin. A urine protein test was positive. A liver biopsy showed chronic hepatitis and positive Congo red staining. Serum and urine immunofixation electrophoresis showed increased monoclonal IgA-κ protein. Color echocardiography and magnetic resonance imaging showed left ventricular wall thickening and slight pericardial effusion. Sanger sequencing showed a heterozygous, autosomal recessive mutation in the *SGPL1* gene (blood). In our patient, amyloidosis was attributed to increased monoclonal IGA-κ protein production by plasma cells after *SGPL1* mutation.

**Diagnosis:**

Primary amyloidosis with multisystem involvement (liver, heart, and kidneys).

**Interventions:**

The patient was administered dipyridamole (25 mg, po, tid), methylprednisolone (40 mg, po, qd, gradually reduced over 6 months), hydroxychloroquine sulfate (200 mg, po, bid), and ursodeoxycholic acid (250 mg, po, tid) until his renal and hepatic markers improved. Immunomodulatory therapy was adjusted according to the patient’s response, and the final regimen was bortezomib injection (1.8 mg, sc, once a week), cyclophosphamide (0.4 g, po, once a week), and dexamethasone (20 mg, po, twice a week), which is currently ongoing.

**Outcome:**

Immunomodulatory therapy improved and stabilized the patient’s condition.

**Lessons:**

*SGPL1* mutation may cause multisystem amyloidosis by disrupting S1P homeostasis and increasing monoclonal IGA-κ protein production, leading to amyloidosis. This case highlights the importance of genetic screening and an improved understanding of *SGPL1* gene mutations.

## 1. Introduction

Systemic amyloidosis is a group of conditions caused by the accumulation of extracellular deposits of insoluble amyloid fibers in multiple tissues and organs. Systemic amyloidosis has a complex and heterogenous presentation, which often leads to a delayed diagnosis. The most common types of amyloidosis are immunoglobulin light-chain amyloidosis (AL), systemic light-chain amyloidosis, and immunoglobulin heavy-chain amyloidosis (AH).^[1,2]^ The AL type is more common than the AH type, with an incidence of 8 to 10 cases per million person-years.^[3]^ Clinically, the various types of immunoglobulins and their light chains can be identified using immunofixation electrophoresis, which is helpful for diagnosis and treatment. These immunoglobulins mainly appear as monoclonal forms, and exist in a partially folded state as their primary structure; they are then further folded and curled to form their secondary structure of β-lamellae, and after binding with aminoglycans and serum amyloid P substance, they transform into an extremely difficult to dissolve and stable amyloid fiber sheath structure, which is deposited in multiple organs, causing organ damage.^[4]^

The relationship between amyloidosis and gene mutation is also a hot topic of research. Significant differences exist in the genetic basis of different amyloidosis subtypes. The AL type is mainly caused by the monoclonal proliferation of plasma cell clones producing monoclonal immunoglobulin light chains, while the AH type originates from heavy-chain abnormalities. Transthyretin amyloid polyneuropathy is an autosomal dominant disease caused by mutation of the transthyretin (TTR) gene, and is mainly characterized by peripheral nerve damage.^[5]^ The pathological mechanism of Alzheimer disease is also related to amyloid, and amyloid deposition is a key step in the progression of Alzheimer disease.^[6,7]^ Caloric restriction and regulation of mammalian target of rapamycin proteins may affect amyloid metabolism and clearance.^[8]^ Although some progress has been made, the relationship between amyloidosis and gene mutations is insufficiently understood, and further discussion and research are needed.

Multisystem amyloidosis associated with sphingosine-1-phosphate lyase 1 (*SGPL1*) mutation is a rare and genetically complex disease, supported by emerging evidence linking SGPL1 dysfunction to aberrant sphingolipid metabolism and amyloidogenesis.^[9,10]^ Epidemiologically, this condition exhibits an extremely low incidence rate (no precise statistics are available due to its rarity, with only sporadic case reports documented globally), and affects patients across a wide age spectrum, ranging from adolescents to elderly individuals. The *SGPL1* gene plays a key role in sphingolipid metabolism, and its mutation can affect the related metabolic pathways, leading to the production and accumulation of abnormal proteins, which then induce amyloid deposition. Serum and urine immunofixation electrophoresis provide a sensitive diagnostic method, while other laboratory abnormalities, including blood routine, liver function, kidney function, and proteinuria tests, can serve as supplementary diagnostic tests.^[9,10]^ The goal of treatment is to reduce the level of monoclonal immunoglobulin light chains in the body, prevent the further deposition of amyloid protein, and reduce or reverse the organ dysfunction caused by amyloid protein deposition.^[11]^ In a recent study, AL patients were treated with autologous peripheral blood stem-cell transplantation and immunotherapy in combination with chemotherapy.^[12]^ The results of the study suggested that although high-dose melphalan combined with stem-cell transplantation is still considered a high-risk treatment for AL, the advent of new agents like bortezomib could potentially transform this treatment paradigm and enhance patient outcomes. Due to its rarity and complexity, the treatment of *SGPL1* gene mutation-associated multisystem amyloidosis remains challenging. Current treatments are mainly aimed at relieving symptoms, delaying disease progression, and improving the quality of life of patients. In the future, with the in-depth study of the pathogenesis of this disease, more effective treatment strategies are expected to be developed.

Here, we report a case of monoclonal IgA-κ amyloidosis caused by an *SGPL1* gene mutation, and review the literature on the potential effects of *SGPL1* gene mutations on various organs, the underlying mechanism of *SGPLI*-related amyloidosis, and the prognosis of patients with this rare disorder.

## 2. Case presentation

### 2.1. Chief complaints

A 53-year-old man was admitted to the Affiliated Hospital of Zunyi Medical University with a 5-month history of abnormal renal function and abdominal distension, which had worsened in the past 2 days.

### 2.2. History of present illness

The patient had been found to have abnormal renal function 5 months ago in Zunyi Medical College Hospital, and had been diagnosed with nephrotic syndrome associated with abnormal liver function. He had been treated with prednisolone tablets, dipyridamole, and hydroxychloroquine sulfate for the nephrotic syndrome, and ursodeoxycholic acid to protect liver function. After comprehensive treatment, his symptoms improved, and he was discharged from the hospital. Then, 2 days ago, he began experiencing abdominal distension that was not related to eating, acid reflux, heartburn, fatigue, or loss of appetite. Consequently, he returned to the Affiliated Hospital of Zunyi Medical University for further treatment.

### 2.3. History of past illnesses

Over 10 years ago, the patient developed severe hydronephrosis secondary to a left renal stone, resulting in the complete loss of left renal function, and consequently, he underwent left nephrectomy at a local hospital. Six months prior, he presented to our institution with bilateral lower extremity edema; diagnostic evaluation by our team confirmed nephrotic syndrome. He completed a course of corticosteroid therapy combined with acid suppression and gastroprotective therapy (such as proton pump inhibitors) and renal supportive care, achieving clinical improvement prior to discharge. Two months earlier, he was diagnosed with essential hypertension (stage 2, very high-risk group) at Southwest Hospital. Although he was prescribed antihypertensive medications, his treatment adherence was suboptimal. Despite this, his blood pressure remained generally well-controlled. There was no history of diabetes mellitus, coronary artery disease, cerebrovascular accident, tuberculosis, viral hepatitis, typhoid fever, drug or food allergies, blood product transfusion, or renal/adrenal-related disorders.

### 2.4. Personal and family history

The patient has been a smoker since 20 years, and smokes 20 cigarettes per day. He denied having an alcohol addiction. He did not have any relevant family history. His immunization status is unknown.

### 2.5. Physical examination

**Figure 1.**
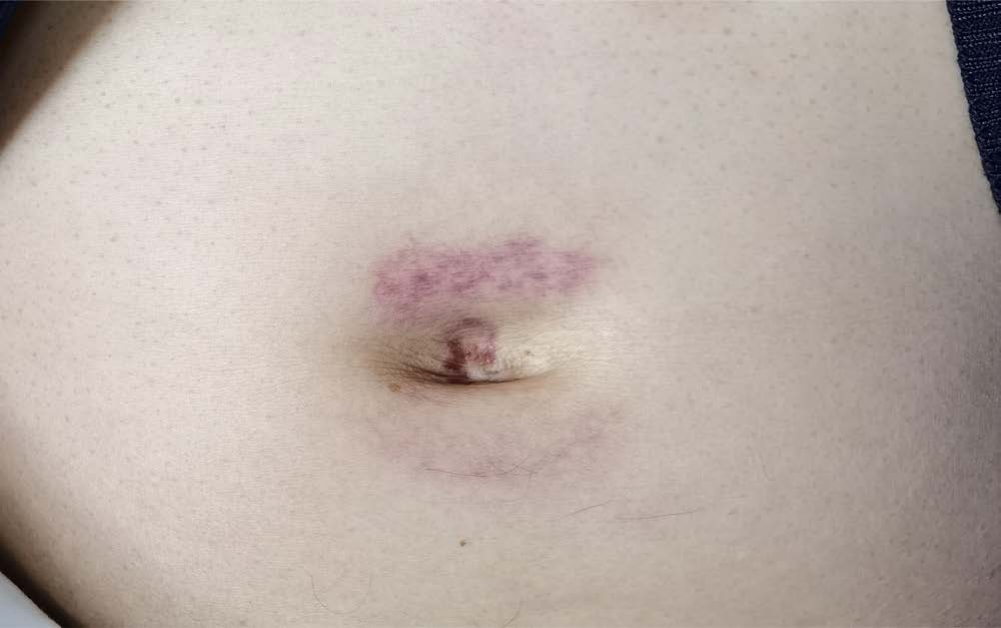
Periumbilical purple erythema.

A physical examination at admission revealed a body temperature of 36.5°C, a pulse rate of 86 beats/min, a respiratory rate of 20 breaths/min, a blood pressure of 115/68 mm Hg, and clear consciousness. The patient had good skin elasticity and yellow skin; he did not have liver palms or spider nevi. He had a purple periumbilical bruise measuring 1 × 3 cm (Fig. 1). Cardiopulmonary examination revealed no obvious positive signs. The abdominal area of the patient was flat, exhibiting tenderness in the left upper abdomen and below the xiphoid process, while showing no rebound tenderness or muscular tension. The patient’s liver was located 5 fingerbreadths below the xiphoid process and 3 fingerbreadths below the left clavicular midline. It was firm in texture, and had sharp edges. There were no palpable nodules or masses. There was slight pain on percussion in the liver area. The test for shifting dullness is negative in this patient. There was no edema in the lower limbs.

### 2.6. Laboratory examinations

Liver-function tests revealed elevated levels of alanine transaminase, aspartate transaminase, alkaline phosphatase, and gamma-glutamyl transferase. The serum albumin level was within the reference range. Kidney-function tests showed increases in the serum levels of urea, uric acid, creatinine, and a decrease in the creatinine clearance rate. Routine blood tests showed an increased white blood cell count and a decreased red blood cell count, platelet count, and hemoglobin level. The dynamic changes in the results of liver-function, renal-function, blood routine, 24-urinary protein, and plasma N-terminal pre-B-type brain natriuretic peptide tests were observed throughout the course of the disease (Tables 1–3). Serum immunoglobulin measurements suggested IgA-κ M proteinemia (Table 4).

**Table 1:** Dynamic changes in biochemical indexes of liver function.

Time	ALT (U/L)	AST (U/L)	TBIL (μmol/L)	GGT (U/L)	ALP (U/L)	ALB (g/L)
Reference range	9–50	15–40	5–21	10–60	45–125	40–55
Before admission to the Infectious Disease Department (November 15, 2021)	19	58↑	19.2	1232↑	500↑	32.9↓
After infection treatment (November 26, 2021)	34	31	9.4	627↑	255↑	25.2↓
After discharge from the Infectious Disease Department (December 01, 2021)	39	28	7.5	588↑	230↑	24.5↓
Before admission to the Hematology Department (January 12, 2022)	62↑	56↑	4.8	1004↑	462↑	22.8↓
After hematology treatment (January 24, 2022)	77↑	34	4.4	1027↑	394↑	26.4↓
After discharge from the Hematology Department (January 28, 2022)	70↑	49↑	6.5	1103↑	367↑	25.4↓
First chemotherapy regimen follow-up (February 4, 2022)	25	50↑	9.1	1202↑	659↑	21.9↓
First chemotherapy regimen follow-up (February 16, 2022)	19	41↑	7.7	1037↑	523↑	21.8↓
Second chemotherapy regimen follow-up (April 20, 2022)	45	63↑	7.5	502↑	721↑	26.8↓
Second chemotherapy regimen follow-up (June 23, 2022)	53↑	72↑	10.6	551↑	639↑	28.5↓
Third chemotherapy regimen follow-up (July 7, 2022)	52↑	47↑	8.8	559↑	422↑	24.3↓
Third chemotherapy regimen follow-up (March 3, 2023)	35	45↑	7.9	859↑	426↑	30.8↓
Fourth chemotherapy regimen follow-up (April 26, 2023)	66↑	57↑	12.6	407↑	399↑	31.2↓
Fourth chemotherapy regimen follow-up (July 14, 2023)	75↑	75↑	9.1	444↑	545↑	26.6↓
Fourth chemotherapy regimen follow-up (December 7, 2023)	68↑	61↑	12.9	787↑	/	/
Fourth chemotherapy regimen follow-up (April 7, 2024)	127↑	79↑	10.7	1053↑	/	/
Fifth chemotherapy regimen follow-up (April 12, 2024)	63↑	46↑	9.8	700↑	416↑	28.5↓
Fifth chemotherapy regimen follow-up (April 21, 2024)	75↑	63↑	8.7	840↑	525↑	39.1↓
Sixth chemotherapy regimen follow-up (April 5, 2025)	61↑	55↑	8.4	393↑	315↑	39.2↓
Sixth chemotherapy regimen follow-up (April 25, 2025)	100↑	58↑	9.8	326↑	/	/
Seventh chemotherapy regimen follow-up (July 2, 2025)	37	40	8.6	316↑	322↑	37.4↓
Seventh chemotherapy regimen follow-up (July 17, 2025)	79↑	46↑	18.1	236↑	271↑	37.9↓
Eighth chemotherapy regimen follow-up (July 28, 2025)	39	33	12	153↑	249↑	27.1↓
Eighth chemotherapy regimen follow-up (August 7, 2025)	47	38	8.1	239↑	247↑	32.8↓
Ninth chemotherapy regimen follow-up (August 17, 2025)	28	30	10.2	232↑	256↑	33.3↓
Ninth chemotherapy regimen follow-up (September 12, 2025)	63↑	41↑	11.4	240↑	/	36.2↓

ALB = albumin, ALP = alkaline phosphatase, ALT = alanine aminotransferase, AST = aspartate aminotransferase, DBIL = direct bilirubin, GGT = gamma-glutamyl transferase, TBIL = total bilirubin.

**Table 2:** Changes in renal function, urinary protein, and NT-ProBNP level.

Time	Urea (mmol/L)	Cr (μ mol/L)	UA (μ mol/L)	GFR (mL/(min × 1.73m^2^))	24-h urine protein (g/24 h)	NT-ProBNP (pg/mL)
Reference range	2.8–7.2	41–109	208–428	85–125	0–0.15	<125
Before admission to the Infectious Disease Department (November 15, 2021)	6.2	130↑	604↑	46.57↓	5.16↑	755↑
After infection treatment (November 26, 2021)	5.3	102	529↑	63.11↓	/	/
After discharge from the Infectious Disease Department (December 01, 2021)	6.6	93	505↑	70.45↓	8.10↑	/
Before admission to the Hematology Department (January 12, 2022)	8.5↑	96	502↑	67.33↓	9.95↑	1840↑
After hematology treatment (January 24, 2022)	15.1↑	139↑	461↑	43.31↓	/	/
After discharge from the Hematology Department (January 28, 2022)	12.4↑	114↑	534↑	54.93↓	/	/
First chemotherapy regimen follow-up (February 4, 2022)	8.6↑	152↑	362	38.79↓	8.12↑	6416↑
First chemotherapy regimen follow-up (March 23, 2022)	/	/	/	/	10.99↑	/
First chemotherapy regimen follow-up (February 16, 2022)	6.5	110↑	408	57.21↓	/	1144↑
Second chemotherapy regimen follow-up (April 20, 2022)	6.4	102	456↑	62.67↓	/	/
Second chemotherapy regimen follow-up (June 23, 2022)	6.2	123↑	574↑	49.83↓	9.27↑	/
Third chemotherapy regimen follow-up (July 7, 2022)	5.2	110↑	/	57.21↓	/	/
Third chemotherapy regimen follow-up (August 30, 2022)	/	/	/	/	5↑	/
Third chemotherapy regimen follow-up (October 3, 2022)	/	/	/	/	7.36↑	/
Third chemotherapy regimen follow-up (March 3, 2023)	6.3	119↑	/	51.26↓	/	/
Fourth chemotherapy regimen follow-up (April 26, 2023)	4.8	125↑	539↑	48.64↓	7.3↑	/
Fourth chemotherapy regimen follow-up (July 14, 2023)	4.4	124↑	/	49.06↓	4.19↑	763↑
Fourth chemotherapy regimen follow-up (December 7, 2023)	5.4	133↑	412	45.13↓	/	/
Fourth chemotherapy regimen follow-up (April 7, 2024)	17.1↑	238↑	/	26.91↓	7↑	/
Fifth chemotherapy regimen follow-up (April 12, 2024)	9.5↑	166↑	/	41.47↓	/	/
Fifth chemotherapy regimen follow-up (April 21, 2024)	9.9↑	222↑	/	29.26↓	/	/
Fifth chemotherapy regimen follow-up (August 20, 2024)	/	/	/	/	6.88↑	/
Fifth chemotherapy regimen follow-up (October 19, 2024)	/	/	/	/	4.23↑	/
Fifth chemotherapy regimen follow-up (November 30, 2024)	/	/	/	/	4.86↑	/
Fifth chemotherapy regimen follow-up (January 4, 2025)	/	/	/	/	6.02↑	/
Fifth chemotherapy regimen follow-up (March 10, 2025)	/	/	/	/	3.05↑	/
Sixth chemotherapy regimen follow-up (April 5, 2025)	13.9↑	222↑	/	29.08↓	/	442↑
Sixth chemotherapy regimen follow-up (April 25, 2025)	13.7↑	236↑	546↑	27.02↓	/	/
Sixth chemotherapy regimen follow-up (June 20, 2025)	/	/	/	/	3.64↑	
Seventh chemotherapy regimen follow-up (July 2, 2025)	11.1↑	218↑	344	29.72↓	/	/
Seventh chemotherapy regimen follow-up (July 17, 2025)	9.6↑	231↑	377	27.72↓	5.31↑	/
Eighth chemotherapy regimen follow-up (July 28, 2025)	7.8↑	201↑	/	32.76↓	/	/
Eighth chemotherapy regimen follow-up (August 7, 2025)	12↑	254↑	/	24.74↓	/	/
Ninth chemotherapy regimen follow-up (August 17, 2025)	9.9↑	217↑	538↑	29.88↓	/	/
Ninth chemotherapy regimen follow-up (September 12, 2025)	12.1↑	232↑	558↑	27.58↓	3.23↑	/

Cr = creatinine, GFR = glomerular filtration rate, NT-ProBNP = N-terminal pre-B-type brain natriuretic peptide, UA = uric acid.

**Table 3:** Changes in the results of blood routine tests.

Time	WBC	RBC	Hb	PLT
Reference range	3.5–9.5 × 10^9^/L	4.3–5.8 × 10^12^/L	130–175 g/L	100–300 × 10^9^/L
Before admission to the Infectious Disease Department (November 15, 2021)	10.88↑	4.72	141	230
After infection treatment (November 26, 2021)	6.45	3.78↓	114↓	191
After discharge from the Infectious Disease Department (December 01, 2021)	7.87	3.84↓	115↓	230
Before admission to the Hematology Department (January 12, 2022)	7.2	3.84↓	116↓	170
After discharge from the Hematology Department (January 28, 2022)	12.08↑	3.9↓	119↓	80↓
First chemotherapy regimen follow-up (Febraury 4, 2022)	7.6	4.22↓	126↓	238
First chemotherapy regimen follow-up (Febraury 16, 2022)	5.64	3.6↓	107↓	253
Second chemotherapy regimen follow-up (April 20, 2022)	5.53	4.49	137	352
Second chemotherapy regimen follow-up (June 22, 2022)	5.58	4.29↓	129↓	179
Third chemotherapy regimen follow-up (July 7, 2022)	6.24	3.98↓	118↓	184
Third chemotherapy regimen follow-up (March 3, 2023)	4.84	4.19↓	122↓	193
Fourth chemotherapy regimen follow-up (April 26, 2023)	5.18	4↓	118↓	135
Fourth chemotherapy regimen follow-up (June 14, 2023)	3.33↓	3.06↓	96↓	124
Fourth chemotherapy regimen follow-up (December 7, 2023)	4.7	3.37↓	107↓	124
Fourth chemotherapy regimen follow-up (April 7, 2024)	8.49	4.96	153	120
Fifth chemotherapy regimen follow-up (April 12, 2024)	3.31↓	3.36↓	106↓	90↓
Fifth chemotherapy regimen follow-up (April 21, 2024)	5.11	4.47	140↓	123
Sixth chemotherapy regimen follow-up (April 5, 2025)	4.01	2.79↓	91↓	85↓
Sixth chemotherapy regimen follow-up (April 25, 2025)	4.19	2.99↓	95↓	117
Seventh chemotherapy regimen follow-up (July 2, 2025)	4.74	3.31↓	102↓	94↓
Seventh chemotherapy regimen follow-up (July 17, 2025)	6.75	3.8↓	117↓	107
Eighth chemotherapy regimen follow-up (July 28, 2025)	2.15↓	2.91↓	98↓	233
Eighth chemotherapy regimen follow-up (August 7, 2025)	6.31	3.72↓	112↓	109
Ninth chemotherapy regimen follow-up (August 17, 2025)	4.17	3.02↓	93↓	102
Ninth chemotherapy regimen follow-up (September 12, 2025)	3.54	3.2↓	99↓	99↓

Hb = hemoglobin, PLT = platelets, RBC = red blood cells, WBC = white blood cells.

**Table 4:** Changes in immunoglobulin levels.

Serum protein electrophoresis	Test value (%)	Reference range (%)	Immunoglobulin quantification	Test value	Reference range
Albumin%	53.18	50.97–63.52	IgG	1.86 g/L	7.00–16.00
α1Globulin%	5.33	2.13–4.56	IgA	2.44 g/L	0.70–4.00
α2 Globulin%	18.49	5.94–11.71	IgM	1.49 g/L	0.40–2.30
β Globulin%	16.13	8.81–14.97	IgD	0 g/L	0.00–0.10
γ Globulin%	6.87	14.05–23.38	IgE	8.62 IU/mL	0.00–100
M Albumin%	-		Kappa	1 g/L	1.70–3.70
			Lambda	0.53 g/L	0.90–2.10
			Kappa/lambda	1.89	

Ig = immunoglobulin.

**Figure 2.**
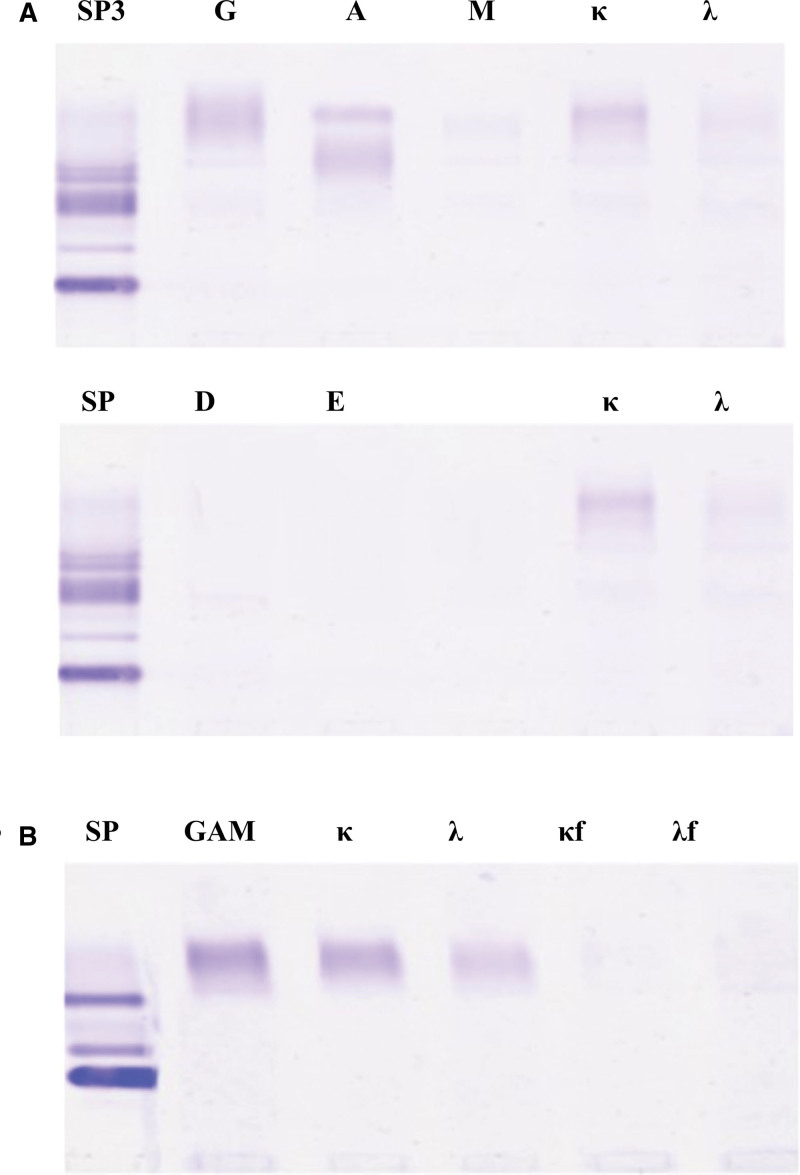
Serum and urine immunofixation electrophoresis results. (A) Serum immunofixation electrophoresis shows M protein bands, which form specific reaction precipitation bands with anti-IgA and anti-kappa. (B) No M protein is observed on urine immunofixation electrophoresis, and no specific reaction precipitation bands were formed with anti-GAM, anti-kappa, anti-lambda, anti-free kappa, and anti-free lambda.

A bone marrow biopsy was performed owing to a suspicion of multiple myeloma, but the results were normal. Immunofixation electrophoresis of serum and urine samples (Table 5, Fig. 2) showed increased levels of monoclonal IgA-κ protein.

**Table 5:** Results of serum free light chain and urine free light chain tests.

Parameter	Results	Reference range
F-κ (blood)	51.20 ↑	3.3–19.4 mg/L
F-λ (blood)	24.48	5.7–26.3 mg/L
F-κ/F-λ (blood)	2.0915 ↑	0.26–1.65
dFLC	26.72 mg/L	
F-κ (urine)	474.93 ↑	0.012–32.71 mg/L
F-λ (urine)	124.52 ↑	0.45–4.99 mg/L
F-κ/F-λ (urine)	3.8141	

dFLC is the difference between F-κ and F-λ, which can be used to assess therapeutic efficacy.

**Figure 3.**
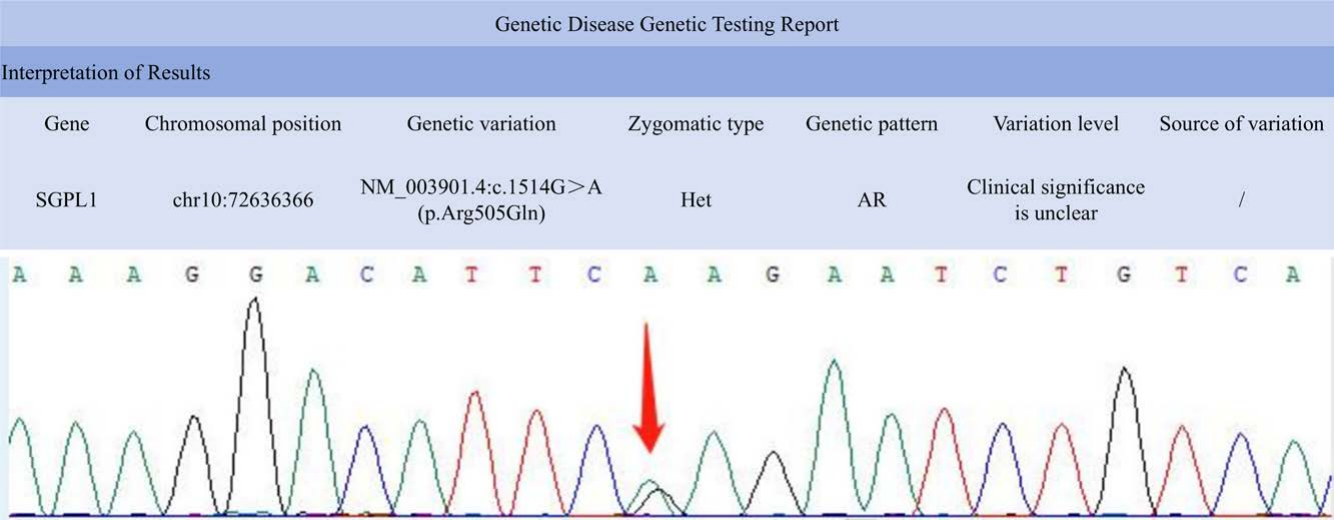
Genetic disease testing shows a heterozygous variation in the *SGPL1* gene, at chromosome position chr10:72636366. AR =autosomal recessive, Het = heterozygous.

Whole exome sequencing for the detection of genetic disorders was performed, and the results revealed a heterozygous, autosomal recessive mutation in the *SGPL1* gene on chromosome 10: c.1514G > A (p. Arg505Gln) (Fig. 3).

### 2.7. Imaging examinations

**Figure 4.**
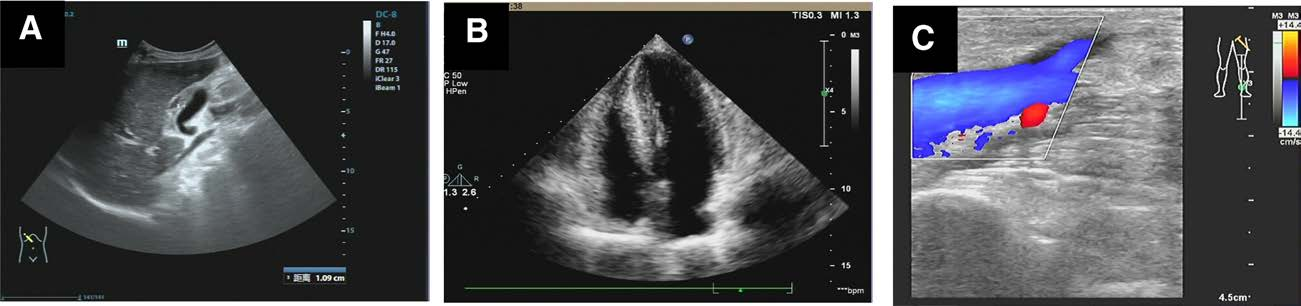
Ultrasonography. (A) Abdominal ultrasonography (October 8, 2021): the oblique diameter of the right lobe of the liver is 158 mm; the anterior–posterior diameter of the left lobe is 112 mm; and the longitudinal diameter of the left lobe is 104 mm. The capsule is smooth. The photoelectric distribution in the liver is uniform, and the distribution of liver blood vessels is clear. The inner diameter of the main portal vein is about 10 mm. The left kidney is not detected (excised), and the right kidney is approximately 125 × 46 mm, with a clear contour and slightly enhanced parenchymal echo. No separation is observed in the right renal collecting system, and CDFI showed abundant blood supply to the right kidney. (B) Cardiac ultrasonography (November 29, 2021): The diameter of each chamber is within the normal range. The left ventricular wall is thickened, and there are no obvious abnormalities in the morphology and structure of each valve. The thickness of the fluid (dark area) in the anterior pericardial cavity is about 3 mm, and the thickness of the fluid (dark area) in the right ventricular pericardial cavity is about 4 mm. Doppler ultrasonography showed mild mitral regurgitation, a forward flow velocity of approximately 180 cm/s in the left ventricular outflow tract, and a peak pressure difference of approximately 13 mm Hg. Mitral valve spectral Doppler shows an E/A ratio < 1. Left ventricular systolic function was as follows: ejection fraction, 74%; fractional shortening, 43%, presenting with congestive heart failure. (C) Bilateral lower limb venous ultrasonography shows no significant abnormalities (February 6, 2022).

**Figure 5.**
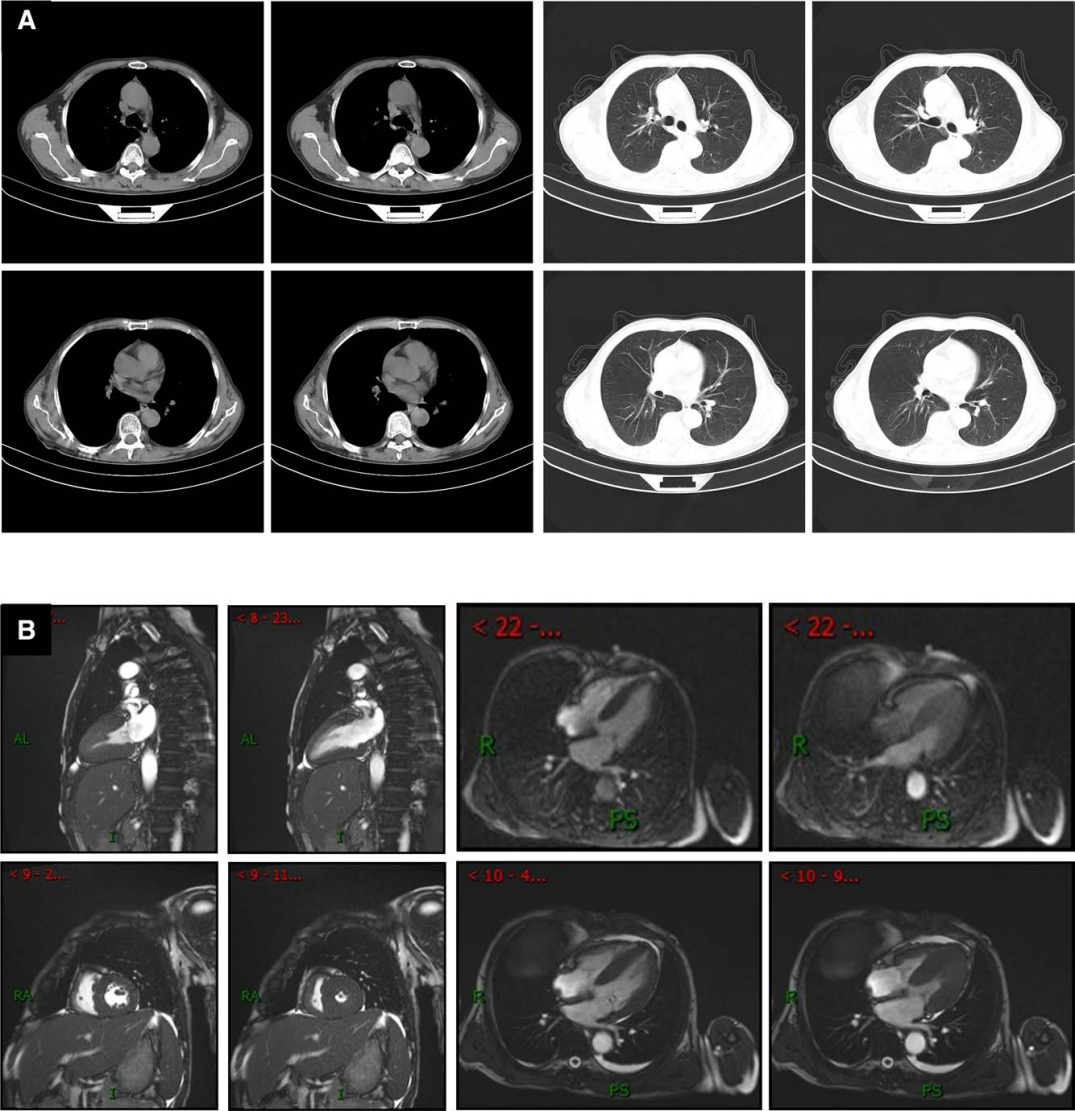
Computed tomography (CT) and magnetic resonance imaging (MRI). (A) Chest CT (November 18, 2021) shows slight streaks and patchy shadows with increased density in both lungs. The trachea and bronchi of each lobe are unobstructed, and the mediastinum is centered. No lymph nodes are found in the mediastinum or hilum. Left ventricular enlargement, and enlargement and decreased density of the liver are seen. (B) Plain and enhanced cardiac MRI scans (February 22, 2022) show uniform thickening of various segments of the left ventricular wall, and linear enhancement shadows around the subendocardial layer of the proximal left ventricle, right atrium, and right ventricular inflow tract, which is consistent with the MRI manifestations of myocardial amyloidosis. A small amount of pericardial effusion, and mild mitral and tricuspid regurgitation are detected. Left ventricular myocardial compliance is decreased, and diastolic function is slightly weakened. Minor pleural effusion is present on both sides.

**Figure 6.**
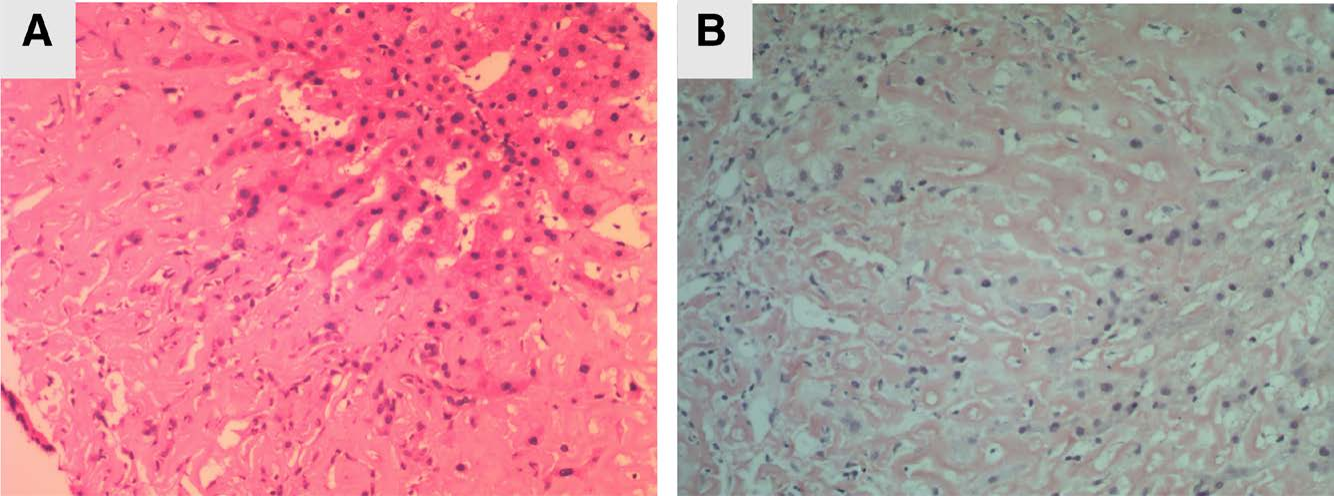
Liver pathology. (A) Liver biopsy tissue with obvious edema and punctate necrosis of liver cells, fibrous tissue proliferation in the portal area accompanied by infiltration of acute and chronic inflammatory cells, which is consistent with chronic hepatitis (G3, S2). Hematoxylin and eosin staining, 40×. (B) Supplementary report: Most of the liver cells and interstitial amyloid changes in the examined tissue are accompanied by interstitial infiltration of acute and chronic inflammatory cells. Congo red staining (+), 100×.

Abdominal color Doppler ultrasonography (Fig. 4A) revealed the following: the liver was enlarged; the gallbladder wall was slightly thickened; the right renal parenchyma was slightly hyperechoic and slightly enlarged; and the left kidney was not detected. Echocardiography (Fig. 4B) showed left ventricular wall thickening and a small amount of pericardial effusion, which are consistent with the ultrasonographic changes of myocardial amyloidosis. Lower extremity venous color Doppler ultrasonography (Fig. 4C) showed no obvious abnormality. A chest computed tomography scan (Fig. 5A) showed bilateral small focal fibrosis in the lungs, mild enlargement of the left ventricle, and enlargement of the spleen and liver with reduced density. Because abdominal color Doppler ultrasonography and chest computed tomography showed liver enlargement, a liver biopsy was performed. The results showed significant edema and punctate necrosis of liver cells, fibrous tissue proliferation in the portal area, and infiltration of acute and chronic inflammatory cells. On Congo red staining (+) of the liver tissue sample, most of the liver cells and interstitial amyloid changes in the examined tissues were observed to be accompanied by the interstitial infiltration of acute and chronic inflammatory cells (Fig. 6). Cardiac magnetic resonance imaging (MRI) was performed to further evaluate the cardiac changes (Fig. 5B), and it revealed the following: uniform thickening of each segment of the left ventricular wall, recent subendocardial changes in the left ventricle, and linear enhancement around the right atrium and right ventricular inflow tract, which are consistent with the MRI findings of cardiac amyloidosis; a small amount of pericardial effusion, and mild mitral and tricuspid valve reflux; decreased left ventricular myocardial compliance, with slightly diminished diastolic function; and slight bilateral pleural effusion.

### 2.8. Final diagnosis

Considering the above findings, we established a final diagnosis of *SGPL1* gene mutation-related multisystem AL with liver, heart, and kidney involvement after left nephrectomy.

### 2.9. Treatment

During the present hospitalization, the patient was diagnosed with nephrotic syndrome, hepatic insufficiency, and status post nephrectomy. Therefore, a series of treatment regimens were administered as follows: 1st, for nephrotic syndrome, dipyridamole tablets were given as anticoagulant therapy at a dose of 25 mg, po, tid, until the nephrotic syndrome stabilized. To delay the progression of kidney disease, methylprednisolone tablets combined with hydroxychloroquine sulfate were used. The initial dose of methylprednisolone was 40 mg, po, qd, which was gradually reduced based on the patient’s condition, with a total treatment course of 6 months. Hydroxychloroquine sulfate was administered continuously at a dose of 200 mg, po, bid, to delay the progression of kidney disease, until the patient’s kidney-function indicators and related symptoms improved. Second, for hepatic insufficiency, ursodeoxycholic acid was given at a dose of 250 mg, po, tid, for liver protection, until the patient’s liver-function indicators and related symptoms improved. During the hospitalization, bone marrow smear and flow cytometry tests showed no abnormal changes. However, echocardiography suggested myocardial amyloidosis. Serum immunofixation electrophoresis revealed an M protein band, which formed a specific precipitation band with anti-IgA and anti-κ. Urine immunofixation electrophoresis showed no M protein, and no specific reaction precipitation band was formed with anti-GAM, anti-κ, anti-λ, anti-free κ, or anti-free λ. Based on the combined results of serum and urine immunofixation electrophoresis, the diagnosis was IgA-κ type M proteinemia. The liver biopsy results indicated significant hepatocyte edema with spotty necrosis, fibrous tissue hyperplasia in the portal area with acute and chronic inflammatory cell infiltration, which is consistent with chronic hepatitis. Congo red staining was positive. Based on the patient’s relevant examinations and clinical symptoms, the diagnosis was confirmed as primary amyloidosis with multisystem involvement (liver, heart, and kidneys). Further chemotherapy was planned, but due to the patient’s poor physical condition, it was postponed until recovery. Therefore, a follow-up was scheduled 1 week after discharge.

### 2.10. Outcome and follow-up

After a definite diagnosis of primary amyloidosis with multisystem involvement (liver, heart, and kidney), the patient was re-admitted for chemotherapy. Based on the patient’s relevant examinations and clinical symptoms, the initial chemotherapy regimen was the BD regimen (January 15, 2022): bortezomib (2 mg, iv, d1, d4, d8, d11) and dexamethasone sodium phosphate injection (20 mg, iv, d1, d2, d4, d5, d8, d9, d12) in combination, with a treatment cycle of 21 days.^[13,14]^ Adjustments were made based on the results of follow-up visits. During the 2nd cycle of the initial chemotherapy regimen, the patient developed symptoms of acute left heart failure. Cardiac MRI confirmed cardiac amyloidosis with decreased ventricular function and poor renal function. Thus, the chemotherapy regimen was changed to the MTP regimen (February 16, 2022): melphalan (2 mg, po, d1, d2, d3, d4, d5, d6, d7), thalidomide capsules (100 mg, po, d1–d30), and prednisone acetate tablets (40 mg, po, d1, d2, d3, d4, d5, d6, d7) in combination, with a treatment cycle of 28 days.^[15]^ Follow-up tests, including blood routine, liver-function, renal-function, 24-hour urinary protein, and cardiac examinations (such as echocardiography), were conducted every 2 to 4 weeks. After 4 cycles of treatment, the patient experienced nausea, vomiting, and discomfort, with decreased renal function and poor treatment response. Hence, the chemotherapy regimen was changed once again, to the VBD regimen (June 27, 2022): bortezomib (1.9 mg, po, d1, d4, d8, d11), lenalidomide capsules (10 mg, po, d1–21), and dexamethasone sodium phosphate injection (20 mg, iv, d1, d2, d4, d5, d8, d9, d11, d12), with a treatment cycle of 28 days.^[16]^ However, after 4 cycles, the patient showed a poor treatment response and severe chemotherapy reactions, leading to the discontinuation of this regimen. A 3rd adjustment was made, and the treatment was changed to the VRD regimen (March 6, 2023): bortezomib (1.9 mg, po, once a week), lenalidomide capsules (25 mg, po, d1–14), and dexamethasone tablets (20 mg, po, twice a week).^[16]^ The patient continued to undergo treatment with the VRD regimen at the outpatient clinic. After the 3rd adjustment to the chemotherapy regimen, the patient developed numbness and pain in his hands and feet during follow-up, and the results of relevant reexaminations were worsened compared to before. Therefore, a fourth adjustment was made: the regimen was changed to the PCD regimen (July 15, 2023): bortezomib injection (1.8 mg, sc, once a week), cyclophosphamide (0.4 g, po, once a week), and dexamethasone (20 mg, po, twice a week). During follow-up, considering the patient’s condition and underlying disease, we had initially planned to treat him with an anti-CD38 monoclonal antibody.^[17,18]^ However, the patient declined due to financial reasons. On April 8, 2025, a 5th adjustment was made, switching to the VRD regimen: bortezomib injection (2 mg, sc, on days 1, 4, 8, and 11), lenalidomide (5 mg, po, on days 1–21), and dexamethasone (10 mg, po, on days 1, 4, 8, and 11). On July 3, 2025, during administration of the VRD regimen, the patient developed skin allergies, prompting a sixth adjustment: the regimen was modified to a weekly schedule consisting of bortezomib injection (2 mg, sc, once weekly), lenalidomide (5 mg, po, on days 1–21), and dexamethasone (10 mg, po, once weekly). On July 29, 2025, the VRD regimen was scheduled again; however, due to prominent injection-site rash following subcutaneous bortezomib administration as well as chest tightness, palpitations, and epigastric distension and pain after lenalidomide intake, the regimen was changed to BD. On August 16, 2025, due to significant gastrointestinal symptoms associated with bortezomib, the treatment was ultimately adjusted to lenalidomide (5 mg, po, on days 1–21) and dexamethasone (20 mg, po, on days 1–21). A repeat test for serum M protein showed that this parameter remained within the normal range (September 24, 2025).

## 3. Discussion

This article reports a case of amyloidosis associated with *SGPL1* gene mutation. The main manifestations of the patient were abnormal liver and kidney function and hepatomegaly. Monoclonal IgA-κ amyloidosis was diagnosed. Immunomodulatory therapy improved and stabilized the patient’s condition. Based on the clinical characteristics of the patient and a literature review, we speculate that this patient had multisystem amyloidosis due to the increased production of monoclonal IgA-κ protein by plasma cells as a result of a mutation in the *SGPL1* gene. This case highlights the importance of genetic screening and an improved understanding of *SGPL1* gene mutations in this disease.

Immunoglobulin light chains can be divided into 2 types: kappa (κ) chains and lambda (λ) chains. The κ and λ chains differ in amino acid sequence and disulfide position.^[19]^ The gene encoding κ chains is composed of the Vκ, Jκ, and Cκ gene clusters.^[20]^ Under physiological conditions, the ratio of κ and λ free light chains in the serum equals 0.26 to 1.65.^[21]^ The κ:λ ratio is altered in conditions associated with the monoclonal proliferation of plasma cells, such as plasmacytoma, and can be used for diagnosis and prognostication. For example,^[22]^ multiple myeloma patients with predominantly λ light chains have a worse prognosis than those with predominantly κ light chains. Moreover, the clinical manifestations of the AL-κ and AL-λ types of amyloidosis are different. In clinical practice, the AL-λ type is more common, and mainly involves the kidneys, whereas the AL-κ type typically involves the gastrointestinal tract and liver.^[23]^ Furthermore, the severity of renal damage differs between the 2 types of amyloidosis: AL-λ type patients with nephrotic syndrome tend to have more severe proteinuria than AL-κ patients, whereas AL-κ patients show higher serum creatinine levels, less amyloid deposition, and a higher degree of renal tubular atrophy and interstitial fibrosis than AL-λ patients, though the specific mechanisms underlying these differences are unknown.^[24–27]^ In the case reported here, the patient was diagnosed with monoclonal IgA-κ amyloidosis by means of serum and urine immunofixation electrophoresis.

The detection of monoclonal κ or λ immunoglobulin light chain deposition in extracellular tissues^[28,29]^ should alert clinicians to neoplastic diseases, such as multiple myeloma.^[30]^ In multiple myeloma, amyloidosis due to λ light chains is more common than that due to κ light chains.^[4]^ Both primary and secondary AL are associated with multiple myeloma, yet they exhibit significant differences in clinical prognosis.^[31]^ Early methods of immunoelectrophoresis and immunofixation were not sensitive enough to detect M protein. At present, the pathogenesis of multiple myeloma complicated with AL is unclear. About 10% of multiple myeloma patients have AL at the time of diagnosis, and another 10% to 15% may go on to develop AL.^[32]^ Compared with the light chains generated in multiple myeloma, the light chains generated in amyloidosis show great stability and are less susceptible to degradation.^[33]^ In clinical practice, the monoclonal IgG-κ type of amyloidosis has the highest incidence, followed by the IgA-κ type, IgM-κ type, and IgD-κ type; biclonal types are relatively rare.^[34]^ Multiple myeloma and AL show similar protein electrophoresis results, but different clinical manifestations: multiple myeloma is clinically characterized by osteodynia, anemia, hypercalcemia, and renal abnormalities, and on diagnostic work-up, abnormalities are often revealed on bone marrow aspiration and flow cytometry, whereas AL primarily affects the viscera. In our patient, a liver biopsy confirmed the presence of hepatic amyloidosis, and a bone marrow biopsy revealed no obvious abnormality in the bone marrow and blood cells, ruling out multiple myeloma. After receiving immunotherapy, this patient exhibited significant clinical improvement. During subsequent regular follow-up periods, the serum M protein level consistently remained within the normal range, with detailed follow-up data presented in Tables 1 to 3. Comprehensive analysis of all follow-up indicators and test results indicates that following treatment with a systemic chemotherapy regimen, the patient’s condition has been effectively controlled and is currently in a stable state.

Although multiple myeloma was definitively ruled out in this patient, the specific cause of his disease remained unknown. Therefore, to further explore the cause and potentially improve the patient’s prognosis, we performed whole exome sequencing for the detection of genetic disorders. The results revealed an *SGPL1* gene mutation. We then reviewed the literature on *SGPL1* gene mutation-related diseases. The *SGPL1* gene encodes the enzyme sphingosine-1-lyase phosphate, which is mainly located in the endoplasmic reticulum of glomerular cells, especially podocytes, mesangial cells, and endothelial cells.^[35]^ Research indicates that homozygous mutations in SGPL1 may lead to a severe, specific type of steroid-resistant nephrotic syndrome.^[36]^ This discovery underscores the central role of *SGPL1* in maintaining the glomerular ultrafiltration barrier, which is a critical renal structure primarily constructed by glomerular podocytes that ensures proper filtration of the blood, while preventing the loss of proteins and other important molecules.^[37]^

The sphingosine-1-lyase phosphate enzyme breaks down sphingosine-1-phosphate (S1P).^[38]^ S1P serves as a ligand for a family of 5 G-protein-coupled receptors (S1P receptors 1–5)^[39]^; the S1P-mediated activation of different combinations of these receptors activates downstream signaling pathways.^[40]^ The biological effects of the S1P signaling pathway may vary depending on differences between cell types.^[41]^ S1P has important regulatory functions in a variety of normal physiological processes as well as disease processes, and mediates a wide range of biological functions.^[42,43]^ By regulating the flow of sphingolipid metabolism, S1P plays a profound role in cellular metabolism and determining cell fate.^[44]^ In endothelial cells, S1P has been demonstrated to enhance endothelial barrier function in a dose-dependent manner, as indicated by measuring transepithelial electrical resistance mediated through S1P receptor-1 and S1P receptor-3.^[45]^ S1P also rapidly promotes the phosphorylation of myosin light chains.^[46]^ The specific Src inhibitor PP2 was found to block S1P-induced FAK phosphorylation and reduce S1P-induced focal adhesion protein translocation to the cell periphery, indicating that S1P modulates FAK phosphorylation via Src activity.^[47]^ Furthermore, research has found that S1P can increase the expression of E-cadherin in intestinal epithelial cells, promote the distribution of E-cadherin and β-catenin in the plasma membrane, and reduce paracellular permeability, thereby enhancing the barrier function of intestinal epithelial cells.^[48]^ Thus, the *SGPL1* gene via its encoded enzyme SGPL1 modulates S1P signaling pathways, and plays an important role in cell and tissue homeostasis.

In this case, we focused on the role of the *SGPL1* gene and its related metabolite S1P in amyloidosis, which is still unclear. By reviewing the literature, we found that SGPL1 deficiency may result in a decreased ability to degrade S1P,^[49]^ thus allowing S1P deposition in the liver,^[50]^ thymus,^[51]^ and other organs. These deposits could eventually lead to visceral involvement in amyloidosis. Therefore, we hypothesized that *SGPL1* gene mutation can lead to the visceral deposition of S1P and subsequent amyloid deposits, leading to viscera-related amyloidosis.

The systemic multi-organ amyloidosis caused by *SGPL1* gene mutation may also be associated with the activation of nucleotide-binding oligomerization domain-like receptor containing pyrin domain 3 (NLRP3) in inflammasomes.^[52]^ The NLRP3 inflammasome complex is a multiprotein complex composed of cytoplasmic NLRP3 sensor molecules, the bridging protein apoptosis-associated speck-like protein, and caspase-1.^[53]^ As an important part of the innate immune system, the assembly and activation of the NLRP3 inflammasome can trigger the release of proinflammatory cytokines.^[54]^ Serum amyloid A is a potent activator of the NLRP3 inflammasome, and activates the NLRP3 inflammasome through cathepsin B, triggering an inflammatory cascade that further activates caspase-1 and leading to the maturation of interleukin-1β, leading to the formation of amyloid fragments.^[55]^ Activation of the NLRP3 inflammasome has been associated with Alzheimer disease.^[56]^ Specifically, *SGPL1* mutations stimulate the astrocyte purinergic receptor P2Y1R through the S1P/S1PR2/4 signaling axis, mediating the activation of the NLRP3 inflammasome, which after assembly, further activates caspase-1 to form amyloid fragments.^[57]^ Therefore, we hypothesized that *SGPL1* mutation could activate the NLRP3 inflammasome and trigger an inflammatory cascade to promote the development of amyloidosis. As discussed above, *SGPL1*-related amyloidosis may represent a rare “inflammation-driven” subtype of amyloidosis, characterized by predominant renal involvement and multisystem manifestations. Its prognosis might be more favorable than that of conventional hereditary subtypes (such as ATTR),^[58,59]^ yet it is crucial to differentiate it from acquired AL-type amyloidosis to prevent misdiagnosis.^[60]^

The *SGPL1* gene has been associated with a variety of diseases, such as steroid-resistant nephrotic syndrome and adrenal insufficiency.^[36]^ Inactivation of the *SGPL1* gene via mutation is associated with sphingosine phosphate lyase insufficiency syndrome, which can lead to congenital nephrotic syndrome, adrenocortical insufficiency, ichthyosis, immune deficiency, and a variety of neurological pathologies.^[61]^ Another *SGPL1* gene mutation has been associated with low calcium levels, primary adrenocortical hypofunction, nephrotic syndrome, subclinical hypothyroidism, lymphopenia, ptosis, and pathological neuroimaging findings.^[62]^ Current research reveals that diverse genetic mutations predispose individuals to multisystem amyloidosis. Pathogenic variants in the *TTR* gene frequently underlie familial amyloidotic polyneuropathy, driven by the aberrant folding and neural deposition of unstable TTR protein.^[63–65]^ Mutations in the apolipoprotein A1 gene correlate with cerebral amyloid angiopathy, compromising cerebrovascular smooth muscle integrity.^[66]^ Gelsolin gene mutations manifest as cranial neuropathy accompanied by corneal opacification,^[67]^ while fibrinogen alpha chain gene alterations precipitate hereditary renal amyloidosis through glomerular damage.^[68,69]^ These mechanisms universally involve misfolded proteins adopting amyloid conformations, ultimately impairing organ function.^[70]^ Notably, our study identified *SGPL1* mutations (distinct from classical amyloid precursors) that induce plasma extravasation and trigger inflammatory cascades, secondarily promoting amyloid deposition. This discovery expands the pathogenic framework of amyloidosis, offering a novel paradigm for precision diagnosis and therapeutic targeting. The above reports emphasize the key role of the *SGPL1* gene in maintaining human health, and show that its mutations can lead to complex pathological changes. A recent study found a significant interplay between sphingosine, S1P lyase, and insulin signaling.^[71]^ These findings of the above study underscore the significance of the close relationship between sphingolipid metabolism and insulin signaling in C2C12 myotubes (known as mouse myoblast cell line), highlighting its potential therapeutic relevance to diabetes mellitus. In addition, *SGPL1* gene mutation has been correlated with the incidence of lung cancer,^[72]^ providing a potential therapeutic target for lung cancer treatment. However, no specific treatment is currently available for *SGPL1* gene mutation-related diseases, and further studies are required to develop effective treatments.

In summary, in this case report, *SGPL1* gene mutation was considered to cause multisystem amyloidosis, based on the results of liver biopsy, serum and urine immunofixation electrophoresis, whole exome sequencing, imaging examination of affected organs, and a literature review. Immunomodulatory therapy stabilized the patients’ condition. Although this article has made a preliminary discussion on the mechanism of *SGPL1* gene mutation-related amyloidosis, further animal model verification and in-depth research on the underlying mechanisms are needed.

## 4. Conclusion

*SGPL1* gene mutation may be associated with multisystem amyloidosis. The underlying mechanism may be that *SGPL1* gene mutation increases S1P production, activates plasma cells, and results in the continuous proliferation of plasma cells and increased secretion of monoclonal IgA-κ protein, which is deposited in various organs, leading to the occurrence of amyloidosis.

## Acknowledgments

We thank *Medjaden* Inc. for the scientific editing of this manuscript.

## Author contributions

**Conceptualization:** Yunfen Chen, Yue Lia, Yinghua Chen, Xiuying Fan, Yihuai He, Yawen Luo.

**Data curation:** Yunfen Chen, Yue Liao, Yinghua Chen, Bangneng Yu, Yihuai He.

**Formal analysis:** Yunfen Chen, Yue Liao, Ya Chen, Xiuying Fan, Xuedong Yi.

**Funding acquisition:** Yihuai He.

**Investigation:** Yunfen Chen, Yue Liao, Mingxia Ding, Ya Chen, Xiuying Fan, Xuedong Yi, Yihuai He, Yawen Luo.

**Methodology:** Yunfen Chen, Yue Liao, Ya Chen, Bangneng Yu, Xiuying Fan, Xuedong Yi, Yihuai He.

**Project administration:** Yihuai He, Yawen Luo.

**Resources:** Yawen Luo.

**Supervision:** Yihuai He, Yawen Luo.

**Validation:** Yunfen Chen, Yawen Luo.

**Writing – original draft:** Yunfen Chen, Yue Liao, Bangneng Yu.

**Writing – review & editing:** Yunfen Chen, Mingxia Ding, Yinghua Chen, Ya Chen, Xiuying Fan, Xuedong Yi, Yihuai He, Yawen Luo.
